# Ophthalmoplegia Caused by Non-Aneurysm Neurovascular Conflict: Clinical Cases of Eight Patients

**DOI:** 10.31083/RN47615

**Published:** 2026-03-12

**Authors:** Xiuyun Kong, Shilei Cui, Jiawei Wang, Hanqiu Jiang

**Affiliations:** ^1^Department of Neurology, Beijing Tongren Hospital, Capital Medical University, 100730 Beijing, China

**Keywords:** neurovascular compression, ophthalmoplegia, abducens nerve, oculomotor nerve, magnetic resonance imaging

## Abstract

**Background:**

Neurovascular conflict (NVC) is a rare but often overlooked cause of oculomotor cranial nerve (OCN) palsy. In this study, we aimed to enhance understanding of NVC as a potential cause of cranial nerve palsies by summarizing the characteristics of eight OCN palsy cases associated with NVC and reviewing previously reported cases.

**Methods:**

A retrospective case series of eight patients with OCN palsy due to NVC was analyzed. Diagnosis was made using 3.0 Tesla magnetic resonance imaging (MRI) with 3D-fast imaging with steady-state acquisition (3D-FIESTA) sequences. Differential diagnoses like myasthenia gravis, inflammatory diseases, and aneurysms were excluded.

**Results:**

The study involved eight patients (six males, two females), aged 34–78 years (average 58.4 years). Six had abducens nerve palsy, and two had partial oculomotor nerve palsy, all exhibiting painless partial ophthalmoplegia. Imaging revealed OCN compression by various arteries: abducens nerve palsy was due to the basilar artery (three patients), vertebral artery (one patient), and anterior inferior cerebellar artery (two patients); two cases of partial oculomotor nerve palsy were caused by compression of the superior cerebellar artery and the posterior cerebral artery, respectively.

**Conclusion:**

NVC should be considered in patients with abducens nerve palsy, particularly those with intermittent symptoms and painless ophthalmoplegia, especially if they have atherosclerotic risk factors; in this descriptive single-center cohort, diagnosis was supported by high-resolution MRI, including 3D-FIESTA and magnetic resonance angiography.

## 1. Introduction

Neurovascular conflict (NVC)-induced oculomotor cranial nerve (OCN) palsy, a specific form of ophthalmoplegia, is an uncommon but clinically significant condition. In evaluating OCN palsy, the primary focus is usually on common causes such as microvascular ischemia, trauma, or cavernous sinus and brainstem lesions due to demyelination, inflammation, or infarction [1, 2, 3]. Priority is often given to identifying compression injuries caused by vascular malformations, aneurysms, or tumors due to their critical need for immediate management. Nonetheless, it is crucial to recognize that even normal arterial loops or tortuous and dilated arteries can cause symptomatic compression, leading to OCN palsy [4, 5, 6].

NVC is a well-established pathogenic factor associated with conditions such as trigeminal neuralgia and hemifacial spasm, both of which are relatively easy to diagnose due to their characteristic episodic pain or spasm [7]. Superior oblique myokymia, characterized by episodic involuntary eye movements, is also linked to NVC. The most common types of OCN palsy caused by NVC involve the oculomotor and abducens nerves [8]. Although treatment options are still debated, pharmacological therapy is generally considered the most suitable first-line approach. In some cases, patients may benefit from decompression surgery.

Due to the lack of aneurysmal abnormalities in morphology and the absence of paroxysmal agitation in symptoms, OCN palsy resulting from non-aneurysmal arterial compression induced by NVC is often disregarded or erroneously diagnosed as ischemic microangiopathy. In this study, we present 8 cases of OCN palsy associated with NVC and drew on relevant published literature to contextualize our findings. Our primary aim is to delineate the clinical features and offer additional insights to enrich the diagnostic process in this domain.

## 2. Methods

Patients with OCN palsy associated with NVC, who were admitted to the Department of Neurology at Beijing Tongren Hospital, Capital Medical University, between January 2022 and December 2024, were consecutively enrolled in this study.

We retrospectively collected in-hospital medical chart records and images of patients with OCN palsy, encompassing involvement of the oculomotor nerve, abducens nerve, or trochlear nerve. Exclusion criteria comprised patients with ophthalmoplegia stemming from infections, strokes, tumors, injuries, aplasia, carotid cavernous fistulae, aneurysms, and intracranial hypotension. To eliminate the possibility of vascular malformations and inflammatory diseases, all patients underwent magnetic resonance imaging (MRI) angiography (MRA) and gadolinium-enhanced MRI scans focused on the cavernous sinus.

NVC identification was accomplished using a 3.0 Tesla MRI scanner (Discovery™ MR750 3.0T, software version DV24.0_R01_1344.a; GE HealthCare, Wauwatosa, WI, USA) employing 3D-fast imaging with steady-state acquisition (3D-FIESTA) sequences. The images were independently reviewed by at least two neuro-radiologists who were blinded to the diagnosis and clinical presentation. Initially, each radiologist interpreted the images independently, following which they discussed their findings to reach a consensus on the final results.

Patients with OCN associated with NVC, confirmed through the aforementioned reviews, underwent assessments to exclude the possibility of myasthenia gravis (MG). These assessments included repetitive nerve stimulation (RNS), single fiber electromyography (SFEMG), neostigmine tests, ice test, and fatigue test. Additionally, MG-related antibodies, including acetylcholine receptor (AChR) antibody, muscle-specific kinase (MuSK) antibody, low-density lipoprotein receptor-related protein 4 (LRP4) antibody, striated muscle protein (Titin) antibody, and ryanodine receptor (RyR) antibody, were assayed in all patients. AChR antibody and MuSK antibody were assessed using radioimmunoassay, Titin antibody via Western Blot, and RyR antibody (EK-H12398, Shanghai EK-Bioscience Biotech, Shanghai, China) and LRP4 antibody (EK-H12439, Shanghai EK-Bioscience Biotech) through enzyme-linked immunosorbent assay. Based on these comprehensive clinical, laboratory, and imaging evaluations, only patients with isolated OCN palsy, in whom inflammatory, neoplastic, ischemic, aneurysmal, and neuromuscular junction disorders were excluded, and who demonstrated direct nerve–vessel contact or compression on high-resolution MRI (3D-FIESTA) without alternative structural explanations, were finally included in the analysis.

Statistical analyses were conducted using SPSS Statistics (version 24; IBM Corp., Armonk, NY, USA). Descriptive variables were assessed for normal distribution using the Shapiro-Wilk test and were presented as means ± standard deviation or medians (interquartile range, IQR). All analyses in this study were descriptive, and no population-level inference was intended.

## 3. Results

**Fig. 1.**
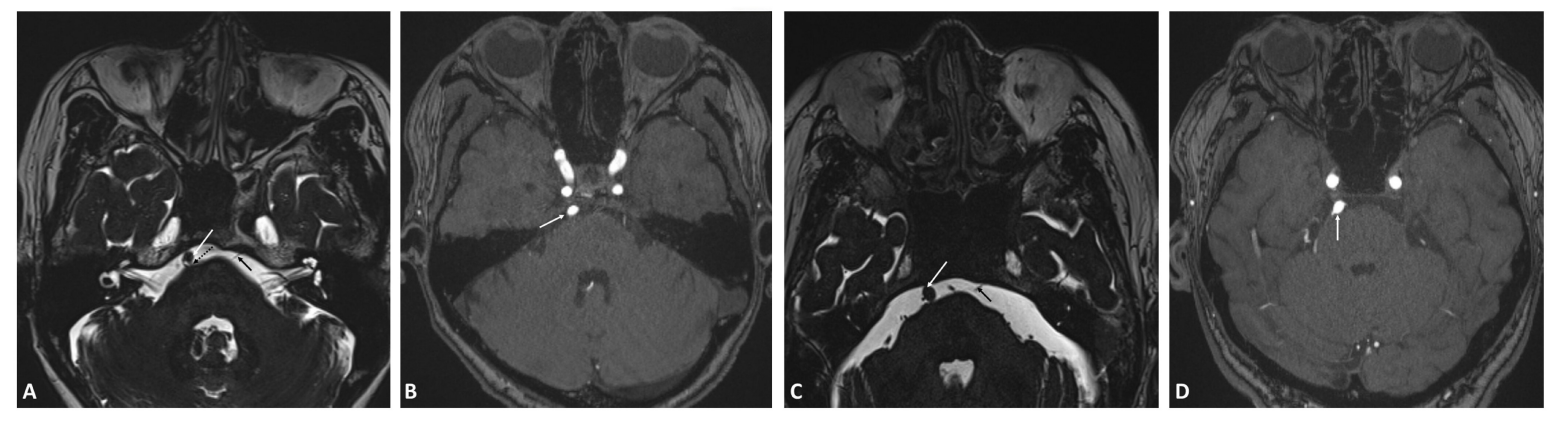
**MRI images of case 1 (A,B) and case 2 (C,D)**. (A) and (C) are the 3D-FIESTA MRI sequence, and (B) and (D) are the original MRA images. In (A) and (C), the cisternal segment of the left abducens nerve (solid black arrow) is clearly visible, with the basilar artery (solid white arrow) located along the pathway of the cisternal segment of the right abducens nerve, resulting in a lack of clear visualization of the cisternal segment of the right abducens nerve. The dashed black arrow in (A) indicates the root of the right abducens nerve in case 1. The original MRA image in (B) and (D) show the basilar artery (solid white arrow). MRI, magnetic resonance imaging; 3D-FIESTA, 3D-fast imaging with steady-state acquisition; MRA, magnetic resonance angiography.

**Fig. 2.**
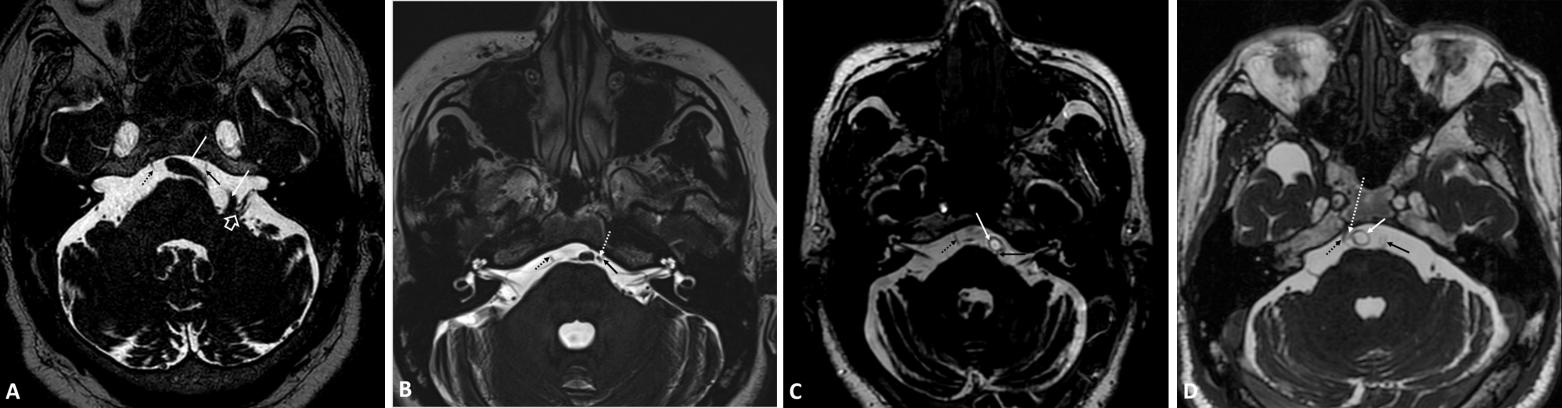
**3D-FIESTA MRI sequence of cases 3 (A), 4 (B), 5 (C) and 6 (D)**. The dashed black arrow indicates the right abducens nerve. In (A), the left abducens nerve (solid black arrow) is located close to the tortuous and dilated left vertebral artery (solid white arrow). Additionally, the left vertebral artery in the left pontocerebellar angle region is tortuous and compressing the brain substance; it is located close to the left VII and VIII cranial nerves. A large white arrow indicates the left VII and VIII cranial nerves. In (B), the left abducens nerve (solid black arrow) appears thinner than the right abducens nerve (dashed black arrow). The left abducens nerve is located close to the anterior inferior cerebellar artery (dashed white arrow). In (C), the left abducens nerve (solid black arrow) is located close to the basilar artery (solid white arrow). In (D), the right abducens nerve (dashed black arrow) is located close to the anterior inferior cerebellar artery (dashed white arrow).

**Fig. 3.**
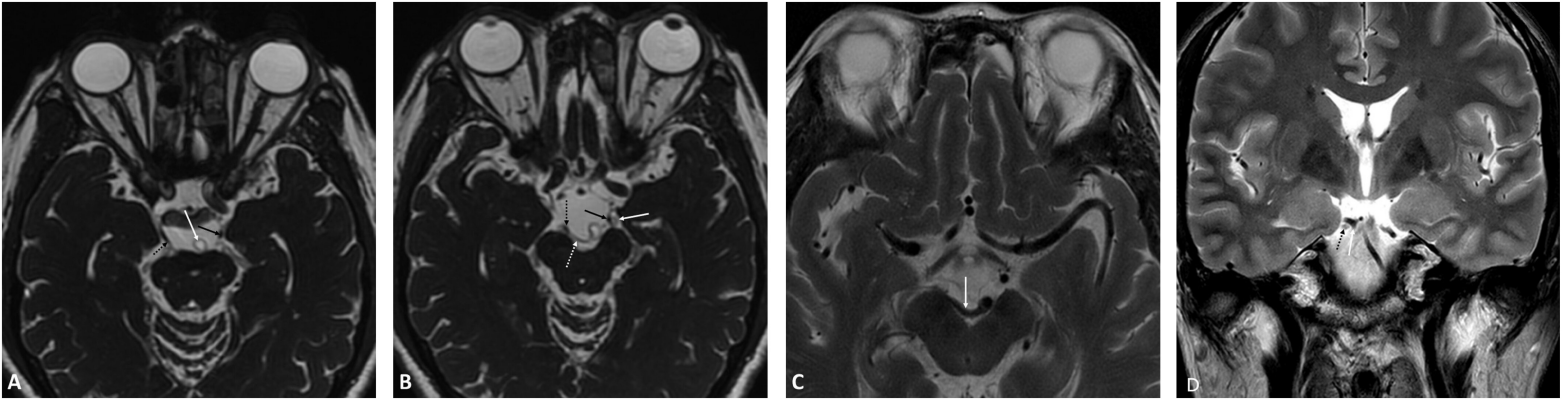
**MRI images of case 7 (A–C) and case 8 (D)**. (A) and (B) are the 3D-FIESTA MRI sequence. The left oculomotor nerve (solid black arrow) and right oculomotor nerve (dashed black arrow) are visualized in (A). The close relationship of the left and right oculomotor nerves with the left superior cerebellar artery (solid white arrow) and right superior cerebellar artery (dashed white arrow) is evident in (B). Furthermore, deformation of the mesencephalon interpeduncular fossa due to compression of the right posterior cerebral artery (solid white arrow) is displayed in (C) (axial T2 imaging). (D) shows the coronal view of the 3D-FIESTA MRI sequence for Case 8, the dorsal aspect of the right oculomotor nerve (dashed black arrow) is closely related to the right posterior cerebral artery (solid white arrow), resulting in arterial elevation and deformation.

A total of 247 patients presenting with OCN palsy were identified, comprising 116 cases of abducens nerve palsy, 77 cases of oculomotor nerve palsy, and 54 cases of trochlear nerve palsy. Upon thorough review of medical records and imaging data, 8 cases of OCN palsy caused by NVC were conclusively diagnosed. Among these cases, 6 were abducens nerve palsy, while 2 cases were partial oculomotor nerve palsy. No instances of trochlear nerve palsy caused by NVC were detected. The occurrence rates of NVC in patients with abducens nerve palsy and oculomotor nerve palsy were 5.2% (6/116) and 2.6% (2/77), respectively. Detailed clinical data of the 8 recruited patients are summarized in Table 1 and Figs. 1,2,3. For the CARE checklist provided in **Supplementary Material**.

**Table 1.** **Summary of clinical features**.

Case NO.	Sex	Age (years)	Disease duration*	Disease course	Symptom duration of first attack	Cranial nerve	Compressing artery	Degree of palsy	Eye pain	RFs of AS
1	F	67	12 y	Recovery followed by relapse	4 w	C VI	BA (Fig. 1A,B)	Partial	No	HL
2	M	78	2 w	Recovery	4 w	C VI	BA (Fig. 1C,D)	Partial	No	HT, HL, smoking, alcohol
3	M	63	2 y	Recovery followed by relapse	1 w	C VI	VA (Fig. 2A)	Partial	No	HT, HL, smoking
4	F	42	15 y	Recovery followed by relapse	3 w	C VI	AICA (Fig. 2B)	Partial	No	altitude sickness, HT
5	M	68	3 mo	Constant	-	C VI	BA (Fig. 2C)	Complete	No	HT, HL, DM
6	M	54	4 w	Recovery	4 w	C VI	AICA (Fig. 2D)	Partial	No	HT, HL
7	M	61	15 y	Constant but fluctuating	-	C III	SCA (Fig. 3A–C)	Partial	No	Hhcy, HL
8	M	34	4 w	Constant	4 w	C III	PCA (Fig. 3D)	Partial	No	Tadalafil

*: disease duration before diagnosis; RFs, risk factors; AS, atherosclerosis; F, female; M, male; y, year; w, week; mo, months; C VI, abducens nerve; C III, oculomotor nerve; NA, not available; BA, basilar artery; VA, vertebral artery; AICA, anterior inferior cerebellar artery; SCA, superior cerebellar artery; PCA, posterior cerebral artery; HT, hypertension; HL, hyperlipidemia; DM, diabetes mellitus; Hhcy, hyperhomocysteinemia.

The average age of the 8 patients was 58.4 years (range: 34–78 years), with 6 males and 2 females. All patients presented with painless ophthalmoplegia, and most had risk factors for atherosclerosis, except for a 34-year-old male patient without any atherosclerotic risk factors who developed isolated right-sided mydriasis the day after taking Tadalafil. The interval from onset to diagnosis varied from 2 weeks to 12 years. Of the six patients with abducens nerve palsy, one exhibited persistent symptom, while the other five showed resolution within 1–4 weeks after the initial episode. However, three of these patients experienced relapses. Among the two patients with partial oculomotor nerve palsy, one presented with pupil sparing, while the other exhibited isolated mydriasis with normal eye movement; neither of these two patients showed symptom resolution. All patients, except for the one who developed symptom after taking Tadalafil, underwent anti-atherosclerotic therapy. This included the use of statins, cessation of alcohol and tobacco consumption, various antihypertensive medications, and heart rate control with beta-blockers. None of the patients underwent microvascular decompression surgery, and cases 2 and 6 demonstrated no recurrence during the 1-year follow-up period. High-resolution MRI demonstrated nerve–vessel contact or compression corresponding to the affected cranial nerve in all eight patients, without alternative structural lesions.

## 4. Discussion

A cranial nerve comprises a segment of the central nervous system and peripheral nervous system connected by a transitional zone, which is particularly vulnerable. This junction contains both myelin sheaths in the central part produced by oligodendrocytes and myelin sheaths in the peripheral part produced by Schwann cells [9]. Given the proximity of the OCN to the vertebrobasilar arterial system, the probability of compression or traction-induced injury at this transitional zone is heightened.

The cisternal segments of the abducens nerve and the oculomotor nerve ascend through the prepontine cistern before traversing the dural cavern surrounded by cerebrospinal fluid. Anatomically, the abducens nerve courses in close proximity to the basilar artery (BA), vertebral artery (VA), and anterior inferior cerebellar artery (AICA), while the oculomotor nerve travels between the posterior cerebral artery (PCA) and superior cerebellar artery (SCA), alongside the BA, and in parallel with the posterior communicating cerebral artery [10, 11]. Consequently, any enlargement or displacement of these arteries, resulting from atherosclerotic deformation or vertebrobasilar dolichoectasia, may directly compress the abducens/oculomotor nerve within the subarachnoid space.

With aging, both the brain and arteries undergo atrophy and atherosclerosis, respectively, to varying degrees, bringing them into closer proximity within a confined space. Due to anatomical characteristics, individuals of Eastern descent tend to have a more crowded cerebellopontine angle compared to other human races [10, 12]. It has been reported that the smaller volume of the posterior cranial fossa may lead to a higher likelihood of vertebrobasilar dolichoectasia, and as patients age and the disease duration increases, the grading of vertebrobasilar dolichoectasia tends to be higher, potentially resulting in an increased likelihood of NVC [13]. Abducens nerve impairment is the most frequently observed, followed by compression of the oculomotor nerve; however, trochlear nerve palsy induced by vascular compression is relatively uncommon [8, 14, 15, 16, 17]. In our study, among the 8 reported patients, 6 had abducens nerve involvement (3 caused by BA compression, 2 by AICA compression, and 1 by VA compression), 2 had partial oculomotor nerve palsy (one due to SCA compression and the other due to PCA compression), and no trochlear nerve impairment was identified. This distribution pattern is consistent with previous reports, which have indicated that the BA is the most frequent offending vessel for abducens nerve palsy caused by NVC (40.9%), followed by the VA (27.3%) and AICA (18.2%) [18, 19, 20, 21, 22]. The offending vessels responsible for oculomotor nerve palsy predominantly include the PCA, posterior communicating artery, SCA, and BA [22, 23].

Although anatomical NVC typically exhibits congenital or chronic progression, resulting ocular motor paralysis often presents acutely due to disturbances in binocular conjugate movements, leading to diplopia. Furthermore, unlike stable compression observed in space-occupying lesions, NVC-induced compression fluctuates due to the dynamic nature of hemodynamics. Consequently, OCN palsy may demonstrate varying courses. Most patients typically develop NVC-induced symptoms in middle to old age, implying that NVC-induced compression may progressively worsen over time. Cross-compression and traction mechanisms only elicit clinical symptoms to a certain extent. Additionally, risk factors such as hypertension, hyperlipidemia, hyperhomocysteinemia, smoking, or alcohol consumption may exacerbate disease progression and contribute to OCN compression. When NVC pressure reaches a critical threshold, an acute onset may occur, potentially triggered by sudden blood pressure elevation. In our study, three cases support this mechanism. In Case 1, symptoms followed prolonged heavy lifting, with fatigue and Valsalva-like maneuvers likely causing transient blood pressure elevation. In Case 4, the onset coincided with altitude sickness and hypertension, with symptoms resolving after blood pressure control. In Case 8, symptom onset occurred after taking Tadalafil, a vasodilator, suggesting that drug-induced vascular changes contributed to the pathogenesis. A previous study has also documented symptom improvement following blood pressure management [21]. It has been reported that the median age of patients with abducens nerve palsy caused by NVC was 55 years (ranging from 11 to 86 years), with hypertension being the most commonly associated condition, followed by hyperlipidemia [21]. Our clinical observations provide practical guidance: painless ophthalmoplegia triggered by acute blood pressure elevation (e.g., heavy lifting, altitude sickness, tadalafil use in Cases 1, 4, 8) is a clinically meaningful clue for NVC.

However, OCN palsy due to ischemic microangiopathy is also an exclusionary diagnosis, typically requiring the exclusion of inflammatory lesions, space-occupying lesions, and MG. Nonetheless, NVC-related OCN palsy is not routinely excluded in clinical practice. Given the similar vascular risk factors, relatively limited cranial nerve symptoms, and benign prognosis, the possibility of underdiagnosed NVC-related OCN palsy should be noted. Ischemic OCN palsy, despite its acute onset with mild to moderate pain, typically follows a monophasic course, with recurrent attacks being relatively rare. Most cases exhibit a recovery period of 2–3 months due to more comprehensive damage. In contrast, all patients with OCN palsy caused by NVC in our study presented with painless onset. Except for one patient with persistent but fluctuating diplopia, all other patients showed a recovery course, and remission after the first episode occurred within 1–4 weeks after onset. The degree of OCN palsy after recurrence was also not complete. The painless onset, intermittent, fluctuating course, and partial palsy of NVC-related OCN palsy have also been reported in previous studies [14, 24, 25, 26, 27]. In a review of NVC-induced abducens nerve palsy, detailed disease information was available for 12 patients, of which 50% had an intermittent disease course, and 50% had a progressive disease course [21]. In another review of NVC-induced oculomotor nerve palsy, 24% of patients had a definite intermittent disease course, and the proportion of patients with intermittent pre-symptoms may be higher, given that both patients and doctors may not pay enough attention to temporary intermittent symptoms [22]. These clinical features may aid in distinguishing between OCN palsy caused by NVC and ischemic microangiopathy.

Ocular MG is typically an important disease to consider clinically in patients presenting with external ocular muscle paralysis without pupil involvement. Early identification and confirmation of the possibility of NVC through neuroimaging can help avoid multiple MG-related tests. Neurovascular conflict should not be regarded solely as a diagnosis of exclusion. 3D-FIESTA MRI provides high-resolution images that enable visualization of both vessels and nerves simultaneously [9]. The diagnosis of NVC requires the support of 3D-FIESTA technology, which imposes high demands on hardware, software, and expertise. Additionally, original images from MRA or thin-slice MRI can provide crucial imaging data [28, 29]. We propose a practical two-step imaging pathway: ① Initial screening with MRA original images (not just synthetic angiograms) to assess vessel-nerve spatial relationships (e.g., basilar artery compression of abducens nerve in Cases 1, 2, 5), as synthetic images only focus on the vascular lumen; ② Confirmatory 3D-FIESTA for suspected cases to obtain quantitative metrics. This balances diagnostic efficiency and accuracy, avoiding unnecessary high-resolution scans.

Asymptomatic cases of NVC in clinical practice present challenges. A prior study utilizing detailed MRI demonstrated that the abducens nerve contacts the AICA in 76.6% of asymptomatic individuals, with the AICA or its main branches penetrating the abducens nerve in 11.4–25.0% of cases [30]. In contrast, the contact rate between the BA or VA and the abducens nerve is notably lower. Therefore, when neuroimaging reveals adjacent compression of the abducens nerve with the BA or VA, the possibility of abducens nerve palsy caused by NVC should be highly suspected.

It is noteworthy that in Case 7, neurovascular compression from the superior cerebellar artery (SCA) likely affected the ventral portion of the oculomotor nerve, sparing the dorsomedial parasympathetic fibers responsible for pupil constriction, thereby resulting in pupil sparing [31, 32]. In contrast, Case 8 exhibited compression of the dorsal aspect of the oculomotor nerve by the PCA, as shown on 3D-FIESTA MRI, likely involving the parasympathetic fibers and causing isolated pupil dilation without extraocular motor involvement. This highlights the importance of compression location in determining clinical presentations.

Based on previous case reports, the majority of patients with OCN palsy caused by NVC have received drug therapy or conservative observation as the primary therapeutic approach. Although microvascular decompression was performed in a small number of cases, which showed complete or partial recovery [21, 22, 33, 34, 35, 36]. Drug therapy primarily focuses on lowering blood pressure and reducing blood lipids, utilizing anti-atherosclerotic medications [21, 22]. Given the possible role of elevated blood pressure and atherosclerosis in pathogenesis, early identification of NVC, management of blood pressure, and other atherosclerosis risk factors may help delay progression and recurrence. Patients in our study received drug therapy but declined surgical treatment, which may be related to their relatively mild symptoms and low acceptance of surgery. Conservative management remains first-line, with individualized trigger control as the core: statins and blood pressure control for atherosclerotic patients, and lifestyle adjustments for those with identifiable triggers (e.g., altitude exposure), aligning with the high spontaneous remission trend in our cohort.

Most prior reports on NVC-induced ophthalmoplegia are limited to isolated case studies, which lack population-based proportion data and systematic analysis of nerve involvement patterns. This study, based on a consecutive series of 247 ophthalmoplegia patients, fills this gap by providing robust single-center epidemiological data: NVC accounts for 5.2% of abducens nerve palsy and 2.6% of oculomotor nerve palsy. Moreover, our finding that abducens nerve involvement (75%) is more frequent than partial oculomotor nerve palsy (25%) aligns with but supplements previous small-sample observations, confirming this distribution pattern in Eastern populations. These data are clinically valuable as they allow clinicians to better estimate the probability of NVC in patients with specific types of ophthalmoplegia, particularly abducens nerve palsy.

Limitations of this study are the small number of cases, potential selection bias and the retrospective nature of the study design, which imposes certain restrictions on the representativeness of clinical characteristics observed in these patients. Furthermore, none of the patients in this study underwent neurovascular decompression surgery, so there is no evidence of post-treatment improvement to support the diagnosis of NVC. However, it must be noted that improvement after surgical treatment is not a necessary condition for the diagnosis of NVC, and not all patients who undergo surgery show improvement, as observed in previous case reports. Quantitative imaging metrics were not applied in this study due to retrospective image acquisition and anatomical variability, in the absence of widely accepted standardized quantitative parameters for neurovascular compression. Nonetheless, compared with previous studies, the advantage of this study lies in its detailed differential diagnosis, particularly in attempting to rule out the possibility of other diseases, such as ocular MG. Further increase in the number of observed cases and prospective cohort studies are warranted to investigate the clinical features and therapeutic benefits in patients with NVC-related ocular motor disorders.

## 5. Conclusion

In conclusion, neurovascular conflict, although rare, should be considered as a possible underlying cause of isolated OCN palsy and should be included in the differential diagnosis in clinical practice. Compressive OCN palsy could arise not only from an aneurysm but also from NVC. Recognizing the diverse presentations and considering non-aneurysmal arterial compression as a potential cause are crucial for accurate diagnosis. Early management of blood pressure and other atherosclerosis risk factors may be helpful. Larger studies and long-term follow-up are warranted to validate the effectiveness of pharmacological interventions and microvascular decompression, and to further refine treatment protocols.

## Availability of Data and Materials

The datasets generated and analyzed during the current study are not publicly available due to privacy concerns. However, the data are available from the corresponding author upon reasonable request.

## Supplementary Material

Supplementary material associated with this article can be found, in the online version, at https://doi.org/10.31083/RN47615.
