# Supplementary material for: Ophthalmoplegia Caused by Non-Aneurysm Neurovascular Conflict: Clinical Cases of Eight Patients
Source: Rev Neurol. 2026 Mar 12;81(3):47615. doi: 10.31083/RN47615 (PMC13036790; doi:10.31083/RN47615)
Supplement: Supplementary file 1 [file 1576-6578-81-3-47615-s1.zip › CARE-checklist-English-2013.pdf]

CARE
case report guidelines

(cc) BY-NC-ND

✔

Topic	Item	Checklist item description	Reported on Line
Title	1	The diagnosis or intervention of primary focus followed by the words "case report"	page 1
Key Words	2	2 to 5 key words that identify diagnoses or interventions in this case report, including "case report"	page 1
Abstract (no references)	3a	Introduction: What is unique about this case and what does it add to the scientific literature?	page 1
	3b	Main symptoms and/or important clinical findings	page 1
	3c	The main diagnoses, therapeutic interventions, and outcomes	page 1
	3d	Conclusion—What is the main "take-away" lesson(s) from this case?	page 1
Introduction	4	One or two paragraphs summarizing why this case is unique (**may include** references)	page 2-3
Patient Information	5a	De-identified patient specific information.	page 2-3
	5b	Primary concerns and symptoms of the patient.	page 2-3
	5c	Medical, family, and psycho-social history including relevant genetic information	page 2-3
	5d	Relevant past interventions with outcomes	page 2-3
Clinical Findings	6	Describe significant physical examination (PE) and important clinical findings.	page 2-3
Timeline	7	Historical and current information from this episode of care organized as a timeline	page 2-3
Diagnostic Assessment	8a	Diagnostic testing (such as PE, laboratory testing, imaging, surveys).	page 2-3
	8b	Diagnostic challenges (such as access to testing, financial, or cultural)	page 2-3
	8c	Diagnosis (including other diagnoses considered)	page 2-3
	8d	Prognosis (such as staging in oncology) where applicable	page 2-3
Therapeutic Intervention	9a	Types of therapeutic intervention (such as pharmacologic, surgical, preventive, self-care)	page 2-3
	9b	Administration of therapeutic intervention (such as dosage, strength, duration)	page 2-3
	9c	Changes in therapeutic intervention (with rationale)	page 2-3
Follow-up and Outcomes	10a	Clinician and patient-assessed outcomes (if available)	page 2-3
	10b	Important follow-up diagnostic and other test results	page 2-3
	10c	Intervention adherence and tolerability (How was this assessed?)	page 2-3
	10d	Adverse and unanticipated events	page 2-3
Discussion	11a	A scientific discussion of the strengths AND limitations associated with this case report.	page 5-6
	11b	Discussion of the relevant medical literature**with references**.	page 3-5
	11c	The scientific rationale for any conclusions (including assessment of possible causes)	page 5-6
	11d	The primary "take-away" lessons of this case report (without references) in a one paragraph conclusion	page 6
Patient Perspective	12	The patient should share their perspective in one to two paragraphs on the treatment(s) they received	No
Informed Consent	13	**Did the patient give informed consent? Please provide if**	Yes√ **No**
